# *Marcks* overexpression in retinal ganglion cells promotes optic nerve regeneration

**DOI:** 10.1038/s41419-024-07281-6

**Published:** 2024-12-18

**Authors:** Xue-Qi Peng, Yan-Zhong Li, Chen Gu, Xuan-Cheng He, Chang-Ping Li, Yong-Quan Sun, Hong-Zhen Du, Zhao-Qian Teng, Chang-Mei Liu

**Affiliations:** 1 Key Laboratory of Organ Regeneration and Reconstruction, Institute of Zoology, Chinese Academy of Sciences, Beijing, China; 2 Savaid Medical School, University of Chinese Academy of Sciences, Beijing, China; 3 Institute for Stem Cell and Regeneration, Chinese Academy of Sciences, Beijing, China; 4 Beijing Institute for Stem Cell and Regenerative Medicine, Beijing, China

**Keywords:** Neuroscience, Brain injuries

## Abstract

Regeneration of injured central nervous system (CNS) axons is highly restricted, leading to permanent neurological deficits. The myristoylated alanine-rich C-kinase substrate (MARCKS) is a membrane-associated protein kinase C (PKC) substrate ubiquitously expressed in eukaryotic cells, plays critical roles in development, brain plasticity, and tissues regeneration. However, little is known about the role of *Marcks* in CNS axon regeneration. Here we show that *Marcks* overexpression promotes robust axon regeneration either before or after optic nerve crush, but insignificantly impacts neuronal survival. Notably, immunostaining and RNA sequencing demonstrate that *Marcks* overexpression does not affect known regeneration-associated genes and pathways. Furthermore, combining CNTF which activates the JAK-STAT3 pathway and *Marcks* overexpression further enhances axon regeneration. Finally, we demonstrate functionally essential effector domain (ED) of MARCKS has similar effects on inducing axon regeneration in RGCs. These results suggest that manipulating *Marcks* and its ED may become a therapeutic approach to promote axon regeneration after CNS injury.

## Introduction

Neurons of the mature mammalian central nervous system (CNS) typically fail to regenerate the injured axons, leading to permanent disabilities (e.g., paralysis after spinal cord injury, blindness after optic nerve injury, coma, or even death after traumatic brain injury) and irreversible neurologic dysfunction of neurodegenerative diseases (e.g., Parkinson’ disease, Alzheimer’s disease). Besides the hostile environment for axonal growth created by CNS myelin and the glial scar, the repair failure of injured CNS neurons is mainly attributed to the insufficient intrinsic regeneration capacity of mature neurons [1–3]. Researches over the past two decades have elucidated some genes controlling the neuronal growth ability, including *Pten*, *Socs3*, CNTF, *Lin28*, *C-myc*, and *Klfs* [4–8]. Nevertheless, none of these gene targets has been applied to clinical. Because each of those molecules partially enhanced the growth ability of mature neurons, in the past few years, a considerable amount of research has been focused on combined treatment strategies [9–11], while many strategies described for promoting neuronal regeneration thus far only facilitated incomplete axonal restoration and partial functional recovery. Therefore, more work is needed to identify key regulatory factors of neural regeneration and provide more effective strategies for CNS regeneration.

The myristoylated alanine-rich C kinase substrate (MARCKS) is a primary target of protein kinase C (PKC), with three highly conserved functional domains [12]. It is bound with the plasma membrane (PM) through its N-terminal myristoylation site and the basic effect domain (ED) in a PKC phosphorylation-reversible manner and becomes an essential regulator of the dynamic actin cytoskeleton, membrane phosphoinositide, and many highly localized molecular interactions, with diverse roles in a variety of cell types, tissues, and organs [13–15]. MARCKS is highly enriched in the nervous system, particularly at the early developmental stage [16, 17]. Genetic studies have shown that MARCKS has multiple roles in the developing nervous system, including neurulation, the formation of the forebrain junction, and the lamination of the cortex and retina [18–20]. In neurons, MARCKS is heterogeneously distributed and enriched in axons and dendritic, and involved in neurite outgrowth, lamellipodia formation, dendrite branching, synapse plasticity, growth cone adhesion and pathfinding, and neuronal migration [12, 21–23]. Most importantly, recent studies have shown that MARCKS is significantly upregulated during optic nerve regeneration and axonal sprouting after brain stroke [24, 25], which indicates that MARCKS is likely to play a vital role in the regeneration process after CNS injury, but this idea still lacks functional research to confirm.

In this study, we first examined how the expression level of *Marcks* in Retinal ganglion cells (RGCs) changed in response to maturation and axotomy in vivo. Next, we explored whether *Marcks* was involved in the regenerative processes of injured RGC axons. Additionally, we performed immunostaining and RNA sequencing in RGCs to gain the molecular mechanisms underlying the *Marcks* for modulating axon regeneration after nerve injury. Finally, by simulating clinically relevant settings, we evaluated the effects of overexpression of *Marcks* and its effector domain (ED) after a delayed post-injury period. Collectively, our results indicate that *Marcks* might be a potential target for facilitating nerve repair, either alone or in combination with other approaches.

## Materials and methods

### Mice

All animal experiments were approved by the Animal Committee of the Institute of Zoology, Chinese Academy of Sciences, and were conducted following the guidelines of national ethical regulations for animal care and use in research (IOZ-IACUC-2021-126). C57BL/6 mice of both sexes at P0, P7, P21, or P56 were used in experiments examining the MARCKS levels in RGCs by aging. In all the other experiments, C57BL/6 mice of both sexes (4–6 weeks) were used. Animals were randomly assigned to each group. All mice were bred in a specific-pathogen-free facility on a 12 h light/dark cycle, and food and water were available ad libitum. All animal surgeries were performed under anesthetized via intraperitoneal injection of Avertin (375 mg/kg, Sigma-Aldrich) diluted in sterile saline.

### Cell lines

Neuro2A cell line (ATCC, stock number: CCL-131) was a kind gift from Dr. Hui Yang. Cells were propagated in DMEM (Gibco) supplemented with 10% fetal bovine serum (Gibco) and 1% penicillin/streptomycin. To test the overexpression efficiency of the *Marcks* construct, Neuro2A cells were first cultured on a 12-well plate to 70–80% confluency. Cells were then transfected with 1 μg *Marcks* construct plasmid by Lipofectamine 3000 for 48 h according to the manufacturer’s protocol.

### Western blot and gene expression analysis

For western blot analysis, cells or tissues were harvested in ice-cold PBS and then lysed in RIPA buffer (Beyotime Biotechnology, P0013B) supplemented with protease inhibitor PMSF (Beyotime Biotechnology, ST506) for 30 min. Cell or tissue lysates were centrifuged at 13,000 rpm for 15 min at 4 °C, and then protein concentrations were quantified using a BCA protein assay kit (Beyotime Biotechnology, P0012). Western blotting was performed according to the standard protocol. The following primary antibodies were used: anti-MARCKS (1:200, Santa Cruz, sc-100777), anti-β-actin (1:3000, Sigma, A5441), and anti-tubulin (1:3000, EASYBIO, BW3312).

Gene expression analysis was performed by RT-qPCR. Total RNA was extracted using Trizol reagent (Invitrogen) and then reverse-transcribed into cDNA using the One-Step gDNA Removal and cDNA Synthesis Kit (Tran, AT311-03). RT-qPCR reactions were performed in triplicate by Hieff^TM^ qPCR SYBR Green Master Mix (YEASEN). *Actb* served as a reference gene. Primers for RT-qPCR are as follows: *Marcks* forward: 5′-TCGCCTTCCAAAGCAAATGGGC-3′, *Marcks* reverse: 5′-TGCCGTTGGCTTGCAGCTCCT -3′.

### AAV construct and AAV packaging

Mouse *Marcks*, CNTF, and human PLAP cDNA were amplified by PCR and then subcloned into the pAAV-EGFP (Addgene, 37825) AAV construct under CAG promoter. AAV plasmids containing the transgenes were co-transfected with pAAV2-RC triple mutant and the pHelper plasmid into HEK293T cells [26]. Seventy-two hours after transfection, the cells were lysed to release the viral particles, which were purified using iodixanol density gradient ultra-centrifugation [27]. q-PCR was used for virus titer measurement. The virus titer was ~10^13^ GC/mL.

### Intravitreal injections for manipulation experiments

For AAV-based experiments, mice were first anesthetized and then injected intravitreally with 1.5 µl of AAV2 virus. The ED peptide (KKKKKRFSFKKSFKLSGFSFKKNKK, 2 µl of 40 µM in 1 × PBS) or the control peptide (KKKNKKKSSLFFSSRFFFKKKGKKK, 2 µl of 40 µM in 1 × PBS) was injected intravitreally at 2 days post crush (dpc). For injections, a pulled-glass micropipette was inserted near the peripheral retina and deliberately angled to avoid damage to the lens. Mice that had intravitreal bleeding or developed signs of inflammation (clouding or an edematous cornea) during or post intravitreal injection were excluded. Exclusion criteria were pre-determined before experimentation.

### Optic nerve crush

We performed optic nerve injury after anesthesia as previously described [8]. Briefly, the optic nerve was exposed intra-orbitally and crushed with forceps (Dumont #5, Fine Science Tools) for 5 s approximately 1 mm behind the optic disc.

### Anterograde tracing of regenerating axons

To assess axon regeneration, the axons were anterogradely labeled by injecting 1.5 µl Alexa Fluor 555-conjugated cholera toxin subunit B (CTB; 1 µg/µl, Thermo Fisher Scientific) via an intravitreal injection 48 h before sacrifice. The mice were transcardially perfused with ice-cold 4% paraformaldehyde (PFA). After perfusion, right optic nerve and bilateral retinas were harvested and post-fixed in 4% PFA overnight at 4 °C. Dehydration and clearing of optic nerves were performed based on previous studies [28, 29]. Briefly, optic nerves were dehydrated in tetrahydrofuran (THF, Sigma-Aldrich), then incubated in benzyl benzoate/benzyl alcohol (BBBA, 2:1 in volume, Sigma-Aldrich) solution until complete transparency.

### Immunostaining

Frozen retinal sections of 20 µm thick were obtained with a cryostat. Whole-mount retinas were radially cut into a petal shape. After being washed with PBS at room temperature for 15 min, sections or whole-mount retinas were immunostained overnight at 4 °C with primary antibody: guinea pig anti-RBPMS (1:200, PhosphoSolutions 1832-RBPMS), rabbit anti-RBPMS (1:500, Abcam ab194213), mouse anti-MARCKS (1:100, Santa Cruz, sc-100777), rabbit anti-pS6 (1:200, Cell Signaling Technology 4858T), rabbit anti-pGSK3β (1:200, Cell Signaling Technology 5558T), rabbit anti-pMARCKS (1:200, Cell Signaling Technology 8722T), rabbit anti-pSTAT3 (1:200, Solarbio K009371P). Then, tissues were incubated for about 2 h at room temperature with Alexa Fluor conjugated secondary antibodies (1:500, Alexa 488, Alexa 555, Alexa 594, Alexa 647, Invitrogen). All antibodies were diluted with PBS containing 0.3% Triton X-100, 2% BSA, and 5% goat serum.

### Microscopy

Z-stacked (step size: 2 µm) and tiled fluorescent images of different experiments (retinal sections, whole-mount retinas, and optic nerve) were obtained with a Zeiss LSM 880 confocal microscope using a 20× or 40× objective. All the images were z-projected to maximal intensity for quantification.

### Image analysis

To analyze the protein level of *Marcks* in RGCs, retinal sections were stained with mouse anti-MARCKS and rabbit anti-RBPMS antibodies following the steps mentioned above (see immunostaining). Fluorescence intensity was measured using ImageJ, and the background fluorescence intensity was subtracted. Only RBPMS^+^ cells were measured.

To analyze S6, GSK3β, STAT3, and MARCKS phosphorylation in RGCs, at least 7 non-adjacent retinal sections from each mouse were analyzed. Fluorescence intensity was measured using ImageJ, and the background fluorescence intensity was subtracted. Only RBPMS^+^ cells were measured.

To quantify the number of regenerating axons in each optic nerve, every 8 consecutive planes were maximum projected to generate a series of 16-µm-thick optical sections. At each 250-µm interval from the crush site, the number of regenerating axons was counted. As described previously [4], the total number of axons per nerve with a radius of r, at a distance d was estimated by summing over all optical sections with thickness *t* (16 µm): Σad = πr^2^ × (average axons/mm)/t.

To quantify RGC survival rate, retina sections or whole-mount retinas were stained with rabbit anti-RBPMS antibody following the steps mentioned above (see immunostaining). We calculated RGC survival rate by dividing the average number of RBPMS^+^ cells in the injured retina (right) by that in the uninjured retina (left).

### RGC enrichment

Retinas were dissected in AMES solution (Sigma A1420, equilibrated with 95% O2/5% CO_2_), digested in EBSS containing papain and DNase (Worthington), and dissociated to single-cell suspensions with manual trituration in ovomucoid solution (Worthington). The dissociated retinal cells were spun down at 450 × *g* for 8 min, resuspended in AMES + 4%BSA to a concentration of 10^5^ cells per ml, and then stained with Thy1.2-PE antibody [30] (1:2000, Invitrogen 12-0902-82) for 15 min at room temperature. After that, cells were washed with an excess of media, spun down, and resuspended again in AMES + 4%BSA to a concentration of 7$\times$10^6^ cells per ml. DAPI was added before filtering cells through a 40-µm cell strainer into fluorescence activated cell sorting (FACS) tubed. High Thy1.2-positive and DAPI-negative cells were collected using Beckman Coulter MoFlo Legacy Cell Sorter with a 100-μm nozzle.

### RGC RNA sequencing and data analysis

RNA-sequencing (RNA-seq) libraries from the purified RGCs were prepared using the Illumina RNA-Seq Preparation Kit. Total RNA sample QC was measured by Agilent 2100 Bioanalyzer (Agilent RNA 6000 Nano Kit). Libraries were sequenced on Illumina NovaSeq 6000 platform. High-quality reads of RNA-seq data were quantified using Salmon (v.1.1.0) with parameters “-i -g --gcBias --validateMappings” [31]. Differential gene expression analysis was generated using DESeq2 (v1.34.0) [32]. Genes with less than 100 counts in total from eight libraries were excluded from analysis, and differentially expressed genes were conducted by *P*-adjust < 0.01, and absolute fold change > 2. Gene set enrichment analysis was performed using clusterProfiler (v4.2.2) [33]. The mouse reference genome sequence (vM24) and gene annotation (vM24) were downloaded from GENCODE (https://www.gencodegenes.org/).

### Statistical analysis

GraphPad Prism 9 was used to generate graphs and for statistical analyses. All data are presented as means ± SEM. Unpaired two-tailed Student’s *t*-tests (two groups) or one-way ANOVA followed by Tukey’s multiple (three or more groups) was performed to determine the statistical significance. Differences were considered significant when *p* < 0.05.

## Results

### *Marcks* is downregulated by aging and injury in RGCs

**Fig. 1:**
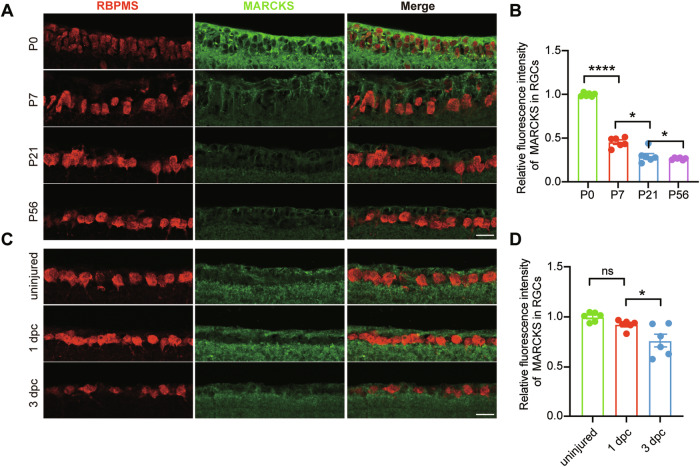
*Marcks* levels are downregulated during development and after optic nerve crush. **A** Representative images of retinal sections from C57BL/6 mice of different ages (0, 7, 21, and 56 days postnatal) showing gradually reduced levels of *Marcks* in RGCs during development. Retinal sections were stained with RBPMS (red), and MARCKS (green). Scale bar: 20 μm. **B** Quantification of fluorescence intensity of MARCKS in RGCs shown in (**A**). ****p < 0.0001, *p < 0.05. RGCs were analyzed from at least 7 non-adjacent retinal sections for each animal, from 6 mice per group. **C** Representative images of retinal sections showing decreased *Marcks* levels in RGCs after optic nerve crush. Retinal sections were stained with RBPMS (red), and MARCKS (green). Scale bar: 20 μm. **D** Quantification of fluorescence intensity of MARCKS in RGCs shown in (**C**). ns no significance, *p < 0.05. RGCs were analyzed from at least 7 non-adjacent retinal sections for each animal, from 6 mice per group.

Both aging and response to injury may mediate the intrinsic growth decline of CNS neurons [5, 34–36]. We postulated that *Marcks* could be involved in the decline and examined the expression of *Marcks* in RGCs at different ages and after axotomy. By performing immunostaining, we found that the expression of *Marcks* protein in RGCs was abundant in young mice but dropped sharply in adults (Fig. 1A, B). Similarly, our unpublished data showed that *Marcks* transcripts in purified RGCs were gradually reduced from P0 to adult (data not shown). We next used an established optic nerve crush (ONC) model to examine the pattern of *Marcks* in response to CNS axon injury. The immunostaining analysis showed that the expression of *Marcks* was unchanged at day 1 after ONC but drastically decreased at day 3 after ONC in C57BL/6 mice (Fig. 1C, D). Besides, the previously published single-cell RNA sequencing dataset showed similar results [37] that the level of *Marcks* in RGCs is decreased after optic nerve injury. Thus, neuron maturation and axonal injury downregulate *Marcks* level in RGCs, which indicates that *Marcks* overexpression may enhance mature CNS neuronal intrinsic growth ability to support axon regeneration after nerve injury.

### *Marcks* overexpression in RGCs induces robust and persistent optic nerve regeneration in vivo

Since *Marcks* is decreased during maturation and after nerve injury, we investigated whether forced overexpression of *Marcks* in RGCs could stimulate optic nerve regeneration in vivo. We first constructed *Marcks*-AAV vector and tested its overexpression efficiency in Neuro2A cells. Western blot results showed that constructed *Marcks*-AAV vectors significantly increased the protein levels of *Marcks* in Neuro2A cells (Fig. S1A, B). Next, we injected AAV virus expressing *Marcks* into the vitreous humor of adult C57BL/6 mice to elevate *Marcks* levels in RGCs. AAV virus expressing placenta alkaline phosphatase (PLAP) was used as the control. Our previous work showed that the virus transduction rate in RGCs was about 89% [38]. We further confirmed successful overexpression of *Marcks* at two weeks after the virus injection in mouse retina tissue (Fig. S2A–C) and RGCs (Fig. S2D–G).

**Fig. 2:**
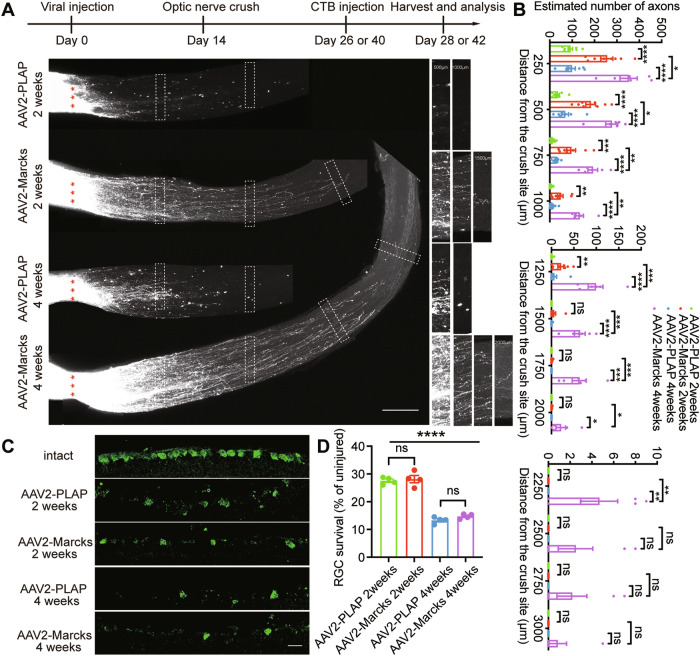
*Marcks* overexpression in RGCs induces significant and persistent optic nerve regeneration. **A** Top: experimental timeline. Bottom: representative image of optic nerve showing that overexpression of *Marcks* in RGCs induced drastic optic nerve regeneration 2 and 4 weeks after optic nerve crush. The right columns showed enlarged images of nerves at 500, 1000, 1500, and 2000 μm distal to the crush sites marked by white boxes on the left. Scale bar: 200 μm. *: crush sites. **B** Quantification of axon regeneration in (**A**). ****p < 0.0001, ***p < 0.001, **p < 0.01, *p < 0.05, ns no significance. *n* = 6–9 mice in each group. **C** Representative images of retinal sections from intact retina or injured retina 2 and 4 weeks after optic nerve crush stained with RBPMS. Scale bar: 20 μm. **D** Quantification of RGC survival in (**C**) showing that *Marcks* overexpression did not affect RGC survival, while fewer RGC survived for 4 weeks after optic nerve crush compared to 2 weeks. ****p < 0.0001, ns no significance. RGCs were analyzed from at least 7 non-adjacent retinal sections for each animal, from 4 mice per group.

To evaluate the functional role of *Marcks* in optic nerve regeneration, we performed ONC 2 weeks after viral injection. We first assessed optic nerve regeneration 2 weeks after the ONC. Axon regeneration was monitored by an anterograde tracer fluorescent-conjugated CTB, which was intravitreally injected 2 days before tissue harvest (Fig. 2A). The fixed optic nerves were tissue-cleared and imaged with confocal microscopy. We found that compared to the control group, in which only a small number of axons were able to cross the crush site, overexpression of *Marcks* significantly increased optic nerve regeneration (Fig. 2A, B). To further explore whether *Marcks* overexpression could induce continued axon regeneration on a longer timescale, we assessed optic nerve regeneration 4 weeks after the ONC. We found that at 4 weeks after ONC, *Marcks* overexpression significantly increased not only the number but also the length of regenerating axons compared with the 2 weeks group (Fig. 2A, B). However, *Marcks* overexpression failed to increase neuronal survival, as revealed by immunostaining retinal sections with antibodies to the pan-RGC marker RBPMS [39] (Fig. 2C, D and Fig. S3A–C). But instead, compared with the 2 weeks group, RGC survival rate significantly declined 4 weeks after the ONC (Fig. 2C, D), indicating that the death of RGCs continued throughout the 4 weeks, and *Marcks* promoted axon regeneration by elevating mature CNS neuronal intrinsic regeneration potential rather than by protecting damaged RGCs. Collectively, these results demonstrated that overexpression of *Marcks* was sufficient to promote optic nerve regeneration up to at least 4 weeks after injury without affecting RGC survival.

### Upregulating *Marcks* in RGCs does not affect PI3K-AKT-mTOR/ GSK3β and JAK-STAT3 pathway

**Fig. 3:**
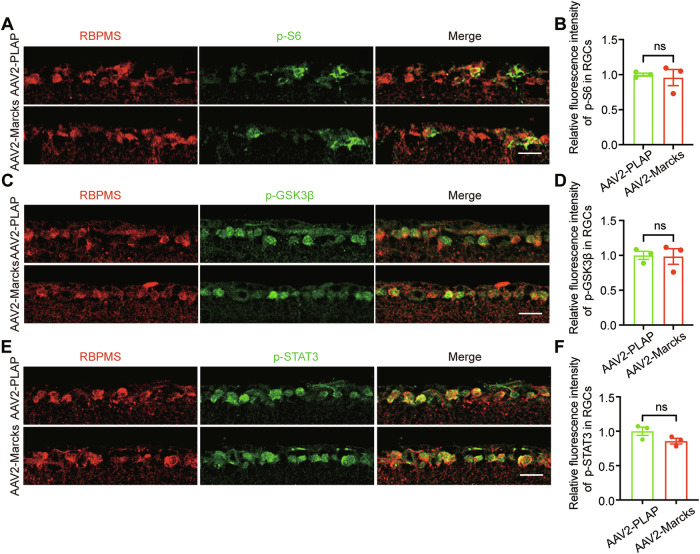
*Marcks* overexpression does not alter the mTOR, GSK3β, and STAT3 pathways. **A** Representative images of retinal sections showing that *Marcks* overexpression did not activate mTOR (marked by p-S6) in RGCs 3 days after optic nerve crush. Retinal sections were stained with RBPMS (red), and p-S6 (green). Scale bar: 20 μm. **B** Quantification of fluorescence intensity of p-S6 in RGCs shown in (**A**). ns no significance. RGCs were analyzed from at least 7 non-adjacent retinal sections for each animal, from 3 mice per group. **C** Representative images of retinal sections showing that *Marcks* overexpression did not inactive GSK3β (marked by p-GSK3β) levels in RGCs 3 days after optic nerve crush. Retinal sections were stained with RBPMS (red), and p-GSK3β (green). Scale bar: 20 μm. **D** Quantification of fluorescence intensity of p-GSK3β in RGCs shown in (**C**). ns no significance. RGCs were analyzed from at least 10 non-adjacent retinal sections for each animal, from 3 mice per group. **E** Representative images of retinal sections showing that *Marcks* overexpression did not increase STAT3 phosphorylation levels in RGCs 3 days after optic nerve crush. Retinal sections were stained with RBPMS (red), and p-STAT3 (green). Scale bar: 20 μm. **F** Quantification of fluorescence intensity of p-STAT3 in RGCs shown in (**E**). ns no significance. RGCs were analyzed from at least 9 non-adjacent retinal sections for each animal, from 3 mice per group.

*Marcks* has been implicated in regulating neuronal migration and axon outgrowth during CNS development by modulating growth cone adhesion and the actin cytoskeleton dynamics [40, 41]. If this is the case, *Marcks* might promote neural regeneration via its effect on the actin cytoskeleton. To test this idea, we examined whether *Marcks* overexpression affected PI3K-AKT-mTOR/ GSK3β or JAK-STAT3, two well-established signaling pathways that promoted RGC axon regeneration [1, 42–44]. We first examined whether PI3K-AKT signaling was altered by *Marcks* overexpression using immunostaining for phosphor-S6 (p-S6) to monitor the activity of PI3K-AKT-mTOR in RGCs and phosphorylation of GSK3β serine 9 residue (p-GSK3β) to monitor the inactivity of PI3K-AKT-GSK3β in RGCs. *Marcks* overexpression did not perturb the activity of mTOR and the inactivity of GSK3β as the immunofluorescence intensity of p-S6 and p-GSK3β remained at the same level as the control (Fig. 3A–D). JAK- and subsequent STAT3 activation is important in initiating axonal growth. To explore whether *Marcks* overexpression was involved in the JAK-STAT3 pathway, we next tested the activation status of STAT3, marked by phosphorylated of its serine 727 (p-STAT3), in *Marcks*-overexpressed RGCs or control RGCs 3 days after ONC. The results showed that the overexpression of *Marcks* did not affect the p-STAT3 level (Fig. 3E, F). Taken together, these results demonstrated that *Marcks* overexpression did not alter PIP3-AKT or JAK-STAT3 signaling pathways to support the intrinsic regenerative ability of RGCs. In addition, unphosphorylated MARCKS directly binds to F-actin or phosphatidylinositol 4,5-bisphosphate (PIP2) to regulate actin dynamic and promote axon outgrowth [15, 41], so we obtained the phosphorylation of *Marcks* pattern 3 days after ONC by performing immunostaining. The results showed that the phosphorylated *Marcks* levels were unchanged (Fig. S4A, B). In conclusion, these results suggested that *Marcks*-regulated neuronal regeneration might rely on unphosphorylated MARCKS which directly regulates actin cytoskeleton.

### *Marcks* overexpression in RGCs does not disturb axon regeneration related program

**Fig. 4:**
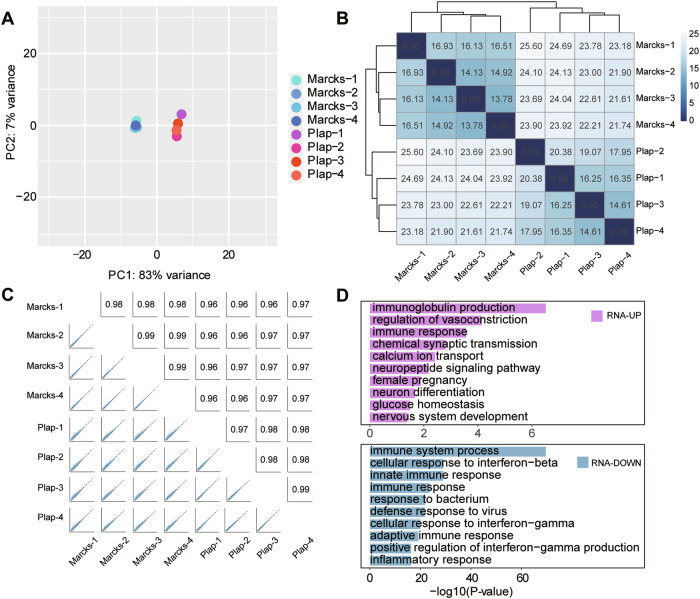
*Marcks* overexpression does not induce changes in transcripts with axon regeneration. **A** Principal component analysis of samples demonstrating the similarity between *Marcks* overexpression and control groups 3 days after optic nerve injury. **B** Hierarchical clustering of samples showing the similarity between *Marcks* overexpression and control groups. The value in each grid represents the Euclidean distance between two samples. **C** Pairwise correlations of samples showing that *Marcks* overexpression had little impact on gene transcription of RGCs. The upper right showed Pearson correlation coefficient between pairwise samples. The lower left gave their scatter plots of normalized counts. **D** GO analysis of differentially expressed genes, the terms with the 10 highest log_10_ (*P*-value) were shown as a bar plot. Results showing that *Marcks* overexpression did not induce changes in transcripts with roles in axon regeneration.

To further investigate the molecular basis underlying *Marcks*-regulated axon regeneration, we performed gene expression profiling analysis in RGCs. To this end, we injected AAV2-PLAP (hereafter called Plap) or AAV2-*Marcks* (hereafter called Marcks) into the vitreous humor of C57BL/6 mice, waited 2 weeks, and performed ONC. Three days later, RGCs were purified from dissociated retinal cells with FACS, and RNA-seq libraries were constructed. Principal component analysis (Fig. 4A), hierarchical clustering (Fig. 4B), and the pairwise Pearson correlations (Fig. 4C) showed that although the 8 samples could be divided into 2 groups consistent with their conditions, each sample was very similar to each other, indicating that gene transcription in RGCs was not significantly affected by *Marcks* overexpression. Meanwhile, from a total of 17103 qualified genes, we identified 888 differentially expressed genes (DEGs) at the threshold of *P*-adjust < 0.01 and absolute fold change > 2, including 125 upregulated and 763 downregulated genes (Fig. S5A and Supplementary Table 1). Using Gene Ontology (GO) analysis, we found that these DEGs were mainly involved in immune response, neuron differentiation, etc., but not particularly in axon growth or regeneration (Fig. 4D). In line with this, GSEA-GO analysis showed similar results: DEGs were enriched in the immune and neurotransmitter processes (Fig. S5B). Furthermore, quantitative transcriptome analysis by RNA-seq showed the signal of the regeneration-associated genes (RAGs) and well-known genes that control axon regeneration [35, 37, 45–47], such as *Lin28a*, *Sox11*, *Pten*, *Socs3*, *Klf4*, etc., was largely unaffected by *Marcks* overexpression (Fig. S5C, D). In addition, we conducted experiments to explore whether *Marcks* overexpression changes the transcriptome level in naïve RGCs. Consistent with our findings in injured RGCs, gene transcription was not significantly affected by *Marcks* overexpression in naïve RGCs (Fig. S6A–D and Supplementary Table 2). In conclusion, these results further supported the view that *Marcks*-induced optic nerve regeneration is unlikely to be through changing the genes or pathways related to axon regeneration.

### CNTF further enhances the axon regeneration-promoting effects of *Marcks* overexpression

**Fig. 5:**
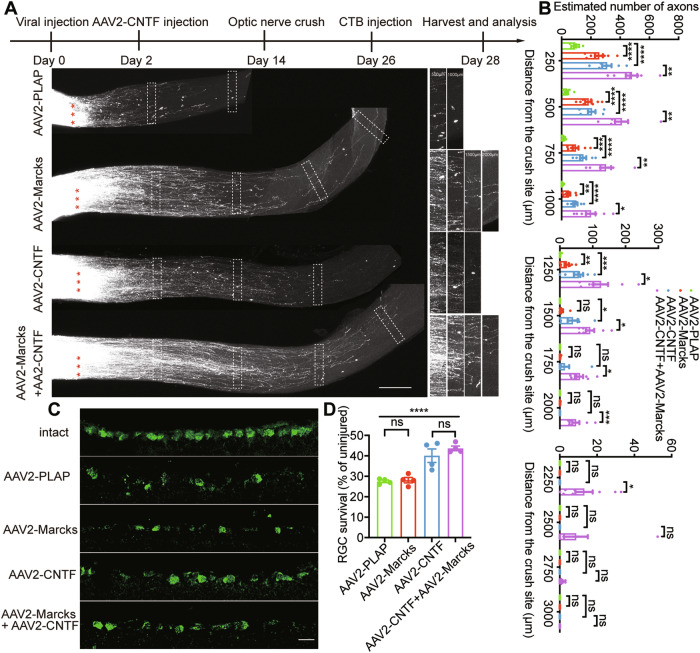
Combinatorial treatment with *Marcks* and CNTF enhances optic nerve regeneration. **A** Top: experimental timeline. Bottom: representative image of optic nerve showing that combining CNTF and *Marcks* overexpression in RGCs produced much stronger axon regeneration 2 weeks after optic nerve crush. The right columns showed enlarged images of nerves at 500, 1000, 1500, and 2000 μm distal to the crush sites marked by white boxes on the left. Scale bar: 200 μm. *: crush sites. **B** Quantification of axon regeneration in (**A**). ****p < 0.0001, ***p < 0.001, **p < 0.01, *p < 0.05, ns no significance. *n* = 7–9 mice in each group. **C** Representative images of retinal sections stained for RBPMS to label RGCs in the intact retina or injured retina for each condition 2 weeks after optic nerve crush. Scale bar: 20 μm. **D** Quantification of RGC survival in (**C**) showing combining *Marcks* and CNTF overexpression in RGCs could not promote a higher RGC survival rate compared with only CNTF expression. ****p < 0.0001, ns no significance. RGCs were analyzed from at least 7 non-adjacent retinal sections for each animal, from 4 mice per group.

Manipulation of *Pten*, *Socs3*, and CNTF enhance RGC survival and regeneration [6, 8]. *Pten* and *Socs3* are endogenous inhibitors of mTOR and JAK/STAT signaling, respectively, and CNTF activates JAK/STAT signaling. However, *Pten*, and *Socs3* are tumor suppressors, and there are risks as treatment targets. Comparatively, CNTF has more application prospects. Our previous results showed that *Marcks* regulated the CNS axon regeneration without affecting JAK-STAT3 signaling pathway, based on these, we then explored whether CNTF and *Marcks* expression have a combinatory promoting effect on optic nerve regeneration. To test this, we injected AAV2-*Marcks* and/or AAV2-CNTF viruses into the vitreous humor of C57BL/6 mice on day 0 and day 2 of the experiment, respectively. Control mice were injected with AAV2-PLAP. The ONC was performed 2 weeks after the viral injection. Two weeks after the ONC, we found that combined CNTF and *Marcks* overexpression resulted in a dramatically increased axon regeneration compared to the single treatment (Fig. 5A, B). Specifically, the combined treatment group induced significantly more robust axon regeneration at different distances (up to 2250 μm) from the lesion boundary (Fig. 5A, B). While overexpression of *Marcks* did not affect RGC survival either without or with additional CNTF compared to respective controls (Fig. 5C, D). These results indicated that the overexpression of CNTF and *Marcks* acted additively in promoting optic nerve regeneration.

### Delayed *Marcks* overexpression still promotes optic nerve regeneration

**Fig. 6:**
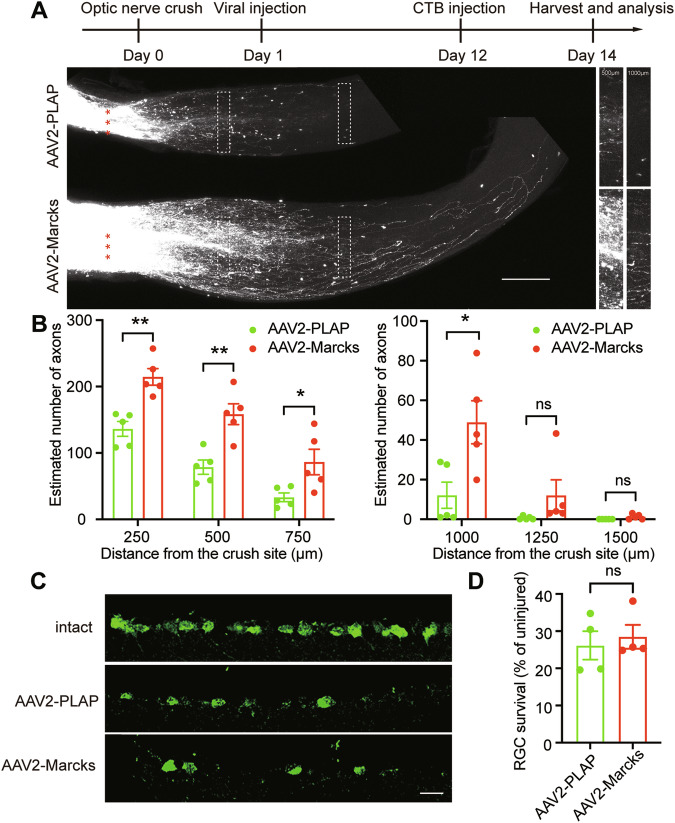
Post-injury *Marcks* overexpression also promotes RGC axon regeneration. **A** Top: experimental timeline. Bottom: representative image of optic nerve showing that post-injury overexpression of *Marcks* in RGCs induced significant optic nerve regeneration 2 weeks after optic nerve crush. The right columns showed enlarged images of nerves at 500, and 1000 μm distal to the crush sites marked by white boxes on the left. Scale bar: 200 μm. *: crush sites. **B** Quantification of axon regeneration in (**A**). **p < 0.01, *p < 0.05, ns no significance. *n* = 5 mice in each group. **C** Representative images of retinal sections from intact retina or injured retina 2 weeks after injury stained with RBPMS. Scale bar: 20 μm. **D** Quantification of RGC survival in (**C**) showing that *Marcks* overexpression did not affect RGC survival. ns no significance. RGCs were analyzed from at least 7 non-adjacent retinal sections for each animal, from 4 mice per group.

To mimic clinically relevant settings, we tested the effects of overexpressing *Marcks* after a delayed post-injury period. We thus injected AAV2-*Marcks* into the vitreous humor of C57BL/6 mice 1 day after optic nerve injury (Fig. 6A). Control mice were injected with AAV2-PLAP. At 2 weeks post-injury, significant numbers of regenerating axons were observed in the mice with delayed *Marcks* overexpression with many regenerating axons growing beyond the lesion border and extending 1 mm from the crush site (Fig. 6A, B). Besides, ONC caused a massive loss of RGCs (~70% loss of RGCs), and there was no significant difference in RGC survival rate between the *Marcks* overexpression group (26.16%) and control group (28.50%) (Fig. 6C, D). Our results suggested that *Marcks* overexpression may be applied to translational practices in treating diseases and injuries involving axon damage.

### *Marcks*’s ED domains play important role in optic nerve regeneration

**Fig. 7:**
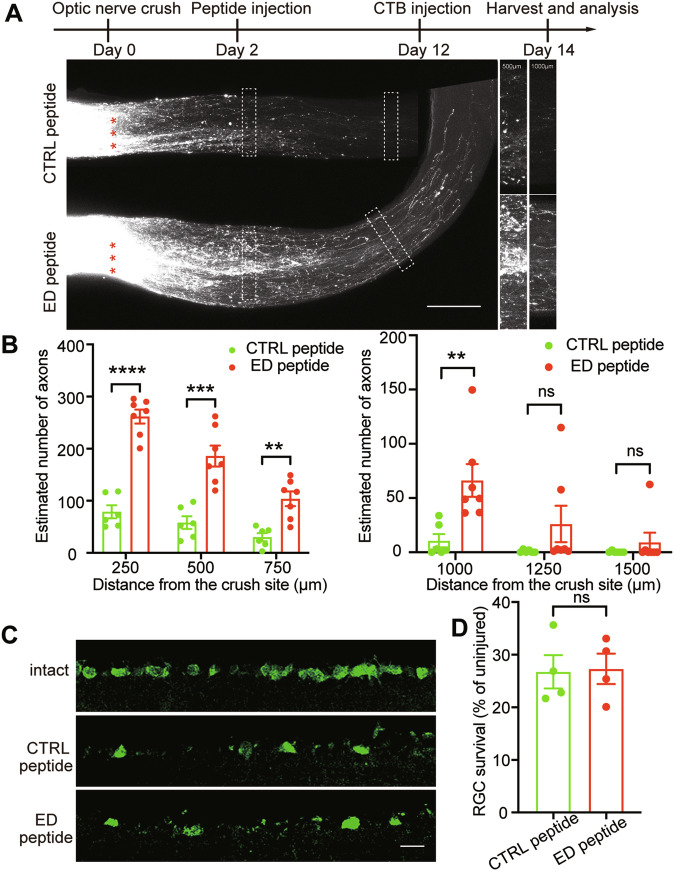
ED peptide stimulates optic nerve regeneration. **A** Top: experimental timeline. Bottom: representative image of optic nerve showing that intravitreal injection of ED peptide stimulated optic nerve regeneration 2 weeks after optic nerve crush. The right columns showed enlarged images of nerves at 500, and 1000 μm distal to the crush sites marked by white boxes on the left. Scale bar: 200 μm. *: crush sites. **B** Quantification of axon regeneration in (**A**). ****p < 0.0001, ***p < 0.001, **p < 0.01, ns no significance. *n* = 6–7 mice in each group. **C** Representative images of retinal sections from intact retina or injured retina 2 weeks after injury stained with RBPMS. Scale bar: 20 μm. **D** Quantification of RGC survival in (**C**) showing that intravitreal injection of ED peptide could not promote RGC survival rate. ns no significance. RGCs were analyzed from at least 7 non-adjacent retinal sections for each animal, from 4 mice per group.

MARCKS protein is an essential protein involved in the coordination of membrane-cytoskeletal signaling events such as cell adhesion, migration, secretion and phagocytosis in various cell types [48]. The most prominent structural feature of MARCKS is a basic effector domain (ED), which binds F-actin, Ca^2+^-calmodulin and acidic phospholipids, and participated in many physiological activities, including regulation of cytoskeleton [15, 41, 48]. Combined with the recent research reported that intravitreal injection of Urocortin rat peptide (41 amino acid peptide, molecular weight 4707.26) can promote RGC survival and axon regeneration after optic nerve injury [37], we synthesized ED peptide (25 amino acid peptide, molecular weight 3080.77), and investigated whether ED peptide could also promote axon regeneration. To test this possibility, we performed the ONC on C57BL/6 mice, intravitreally injected ED or CTRL peptide at 2dpc, anterogradely labeled RGC axons by intravitreal injection of fluorescently conjugated CTB at 12dpc, and counted labeled axons at 14dpc (Fig. 7A). The results showed that the number of axons crossing the lesion border in the CTRL peptide group was minimum. In contrast, ED peptide induced marked CTB fluorescent labeled regenerating axons up to 1 mm from the crush site (Fig. 7A, B). We also assessed RGC survival in the retina using RBPMS as a marker. As expected, the ED peptide, like *Marcks*, did not improve RGC survival (Fig. 7C, D). Besides, we constructed *Marcks*-AAV vector without ED domain (hereafter called *Marcks* ΔED), and we injected AAV virus expressing *Marcks* ΔED into the vitreous humor of adult C57BL/6 mice to explore whether ED domain is critical for *Marcks* function in neural repair. The ONC was performed 2 weeks after the viral injection. The results suggested that *Marcks* without ED domain does not promote axon regeneration 2 weeks after optic nerve crush (Fig. S7A, B). These findings showed that ED-function instead of other domains function is more critical for axon regeneration, meanwhile, therapeutic administration of ED peptide can stimulate axon regeneration after CNS injury and provided a new idea that peptide could become an effective method to repair CNS damage.

## Discussion

Long-distance axon regeneration and meaningful functional recovery are almost nonexistent in the mature mammalian CNS and are also highly challenging therapeutic targets. The combinatorial approaches via modulating multiple independent pathways [5, 9, 11] (e.g., *Pten*/*Socs3* co-deletion, inflammation/cAMP/*Pten* knockout, or *Rheb1* overexpression/visual stimulation) are known to produce robust axon regeneration and reform functional synapses with their original targets, but function recovery remains difficult to achieve, demonstrating that additional studies are necessary for a more accurate view of the mechanisms and synergistic effects of molecules to promote functional CNS repair. In this study, we used the optic nerve injury, an effective and widely used experimental CNS injury model [49], to provide clear evidence that the expression of *Marcks* is significantly decreased during neuron maturation and after optic nerve lesion, and overexpression of *Marcks* is sufficient to enhance optic nerve regeneration. These results again indicate that the ability to regenerate axons deteriorates as neurons mature, and consistent with previous studies [35, 50], modulating these developmentally downregulated genes could raise the regenerative potential in adult neurons.

MARCKS is found associated with the PM. Upon phosphorylation by PKC or calcium-dependent calmodulin-binding, MARCKS translocate from the membrane into the cytosol, thus participating in multiple physiological and cellular functions [12, 48]. MARCKS has been proven to participate in several different molecular interactions depending on its phosphorylation state. Firstly, when MARCKS is unphosphorylated and attached to the PM, it can directly bind F-actin to realize actin filament cross-linking and promote actin polymerization [15]. Since dynamic regulation of the actin cytoskeleton plays a central role in neuronal function in the brain, MARCKS has been implicated in neural development, physiology, and neurodegeneration [12]. The unphosphorylated MARCKS direct interaction with Rab10 in the plasmalemma precursor vesicles provides membranes for the outgrowth of axons [21]. In addition, when MARCKS is not phosphorylated, MARCKS sequesters PIP2 in an area of the PM to promote axon growth, which has been proposed that sequestration of PIP2 by MARCKS regulated cytoskeleton [41]. In contrast, after the phosphorylation of MARCKS, PIP2 becomes accessible to phosphoinositide 3-kinase (PI3K) and phosphorylated to form PIP3, thus affecting the AKT signaling pathway [51]. Finally, phosphorylated MARCKS binds to TOB2 activating ErbB2 signaling [52]. Based on these reports, we tested the phosphorylation level of MARCKS to explore how *Marcks* participated in the neural regeneration process. Our results clearly showed that the phosphorylation level of MARCKS does not change in *Marcks*-overexpressed RGCs at 3dpc, which indicates that the upregulated MARCKS protein is still tethered to the membrane interacting with molecules to play its neural repair function. We further examined two well-known signaling pathways to promote axon regeneration, JAK-STAT3, and PIP3-AKT-mTOR/ GSK3β. The results showed that overexpression of *Marcks* has no effect on these two pathways. Another possibility, MARCKS could be the downstream effectors of these pathways relating to axon regeneration. To test this possibility, future studies, such as, knockout MARCKS in PTEN KO mice, GSK3β KO mice, or STAT3 overexpression mice are needed to elucidate whether MARCKS is required for axon regeneration induced by these pathways.

Further, we performed an RNA-seq to explore intrinsic molecular mechanisms by which *Marcks* overexpression can promote axon regeneration in vivo. Interestingly, we found that all 8 samples were very similar to one another. It is worth noting that there are still 888 differential expressed genes in the two groups, and this may be because we obtained too few purified RGCs and amplified the RNA libraries, many slight differences, especially immune response due to the complexity of surgeries, including ONC and AAV injection, were enlarged. Due to the high Pearson correlation, these differences became significant. However, these processes do not affect the axon regeneration-associated GO terms. More importantly, *Marcks* overexpression has little effect on many known genes to support neural regeneration [35, 45–47] (e.g., *Klf4*, *Lin28a*, *Sox11*). Moreover, Rab10 mRNA level was not significantly changed in our RNA-seq data, indicating that the overexpression of *Marcks* in adult RGCs does not regulate membrane targeting of Rab10 vesicles to promote axon growth. In addition, recent studies have proposed unmasked PIP2 interacts with and inhibits proteins promoting actin dynamics (e.g., gelsolin, cofilin, profilin), thereby indirectly stabilizing the actin cytoskeleton. After sequestration of PIP2 by MARCKS, these proteins are released and promote cell motility [41, 48]. Nevertheless, in our RNA-seq data, the mRNA level of these molecules that affect the cytoskeleton has not changed (Supplementary table 1). Combined with previous immunofluorescence staining experiments, we believe that *Marcks* directly binds to F-actin, affecting cytoskeleton and actin dynamics to participate in the CNS repair process. However, it is a pity that there is no direct evidence to prove this view in this study due to the lack of corresponding cytoskeleton experimental technology for the ONC model in vivo.

MARCKS binds to the PM via the dual action of a hydrophobic, myristoylated N-terminus, and a polybasic stretch within the so-called ED. The phosphorylation at ED dissociates MARCKS from F-actin and results in loss of actin cross-linking and polymerization activity [15, 41, 53]. Therefore, we synthesized a small molecule ED peptide and tested its role in CNS repair. Our results showed that ED peptide significantly promotes axon regeneration after ONC. Recent studies reported that a PTEN antagonist peptide is beneficial in experimental spinal cord injury models [54]. Urocortin rat protein has been shown to promote RGC survival and axon regeneration after optic nerve injury [37]. Additionally, peptide-based inhibitors are expected to become candidate drug molecules for the treatment of Alzheimer’s disease, Parkinson’s disease, and other neurodegenerative diseases [55]. Some of these peptides have been proven to reduce brain injury and alleviate cognitive [56]. Together, our work adds another strong evidence for the application of small molecular peptides in neural repair. It is worth noting that the molecular weight of the ED peptide we synthesized is smaller than that of Urocortin rat previously reported [37], thus in theory easier to be absorbed. However, due to the absence of ED antibody and the sharp increase in molecular weight of fluorescent labeling, we still have insufficient evidence to prove that ED, like AAV virus, infects RGC to play a role in neural repair.

In conclusion, we prove that *Marcks* is an important target molecule for CNS repair and that its overexpression has great therapeutic potential for neurological disorders. Meanwhile, we find that ED plays a key role in *Marcks*-regulated CNS axon regeneration. ED has the general advantages of peptide drugs, such as the potential for high specificity and low toxicity, the possibility of reasonable design, the better chance to cross the blood-brain barrier, the low accumulation in tissues, and the potential of increasing target affinity through modification in the peptide sequence. At present, there are more than 60 therapeutic peptides on the market, about 140 are in clinical trials, and at least 500 are in preclinical evaluation [57]. Therefore, ED has vast clinical potential in CNS repair.

## Supplementary information

Supplemental data

Supplemental table 1

Supplemental table 2

Original Western blotting

## Data Availability

The original RNA-seq data from this study are available in the Genome Sequence Archive (GSA) of BIG Data Center, Beijing Institute of Genomics (BIG), Chinese Academy of Sciences with accession number CAR009731.
