# Supplementary material for: Marcks overexpression in retinal ganglion cells promotes optic nerve regeneration
Source: Cell Death Dis. 2024 Dec 18;15(12):906. doi: 10.1038/s41419-024-07281-6 (PMC11655864; doi:10.1038/s41419-024-07281-6)

***Marcks* Overexpression in Retinal Ganglion Cells Promotes Optic Nerve Regeneration**

Xue-Qi Peng^1,2,3,4*^, Yan-Zhong Li^1,2,3,4*^, Chen Gu^1,2,3,4^, Xuan-Cheng He^1,3^, Chang-Ping Li^1,2,3,4^, Yong-Quan Sun^1,2,3,4^, Hong-Zhen Du^1,3^, Zhao-Qian Teng^1,2,3,4*^, Chang-Mei Liu^1,2,3,4*^

1. Key Laboratory of Organ Regeneration and Reconstruction, Institute of Zoology, Chinese Academy of Sciences, Beijing 100101, China

2. Savaid Medical School, University of Chinese Academy of Sciences, Beijing 100049, China.

3. Institute for Stem Cell and Regeneration, Chinese Academy of Sciences, Beijing 100101, China.

4. Beijing Institute for Stem Cell and Regenerative Medicine, Beijing 100101, China

*These authors contributed equally.

*****Correspondence author: Z-Q.T. ([tengzq@ioz.ac.cn](mailto:tengzq@ioz.ac.cn)), C-M.L. ([liuchm@ioz.ac.cn](mailto:liuchm@ioz.ac.cn))

**Figure legend**

**Figure S1. Validation of *Marcks* overexpression efficiency in Neuro2A cells.**

1. A representative western blot showing increased MARCKS protein level in Neuro2A

cells two days after transfection with *Marcks*-AAV.

1. Quantification of protein level of MARCKS in (A). * p<0.05, n=3 independent

experiments.

**Figure S2. Verification of *Marcks* overexpression in RGCs.**

1. *Marcks* mRNA levels were increased in mouse retina tissues infected with AAV2-

*Marcks* compared with the control tissues infected with AAV2-PLAP. ** p<0.01, n=4 independent experiments.

1. A representative western blot showing increased MARCKS protein level in the retina

two weeks after intravitreal injection AAV2-*Marcks*.

1. Quantification of protein level of MARCKS in (B). * p<0.05, n=3 independent

experiments.

1. Representative images of immunostaining of sectioned retina with RBPMS

(green) and MARCKS (red). Note the markedly increased MARCKS staining in RGCs 2 weeks infected with AAV2-*Marcks* compared to that infected with AAV2-PLAP. Scale bar: 20 μm.

1. Quantification of fluorescence intensity of MARCKS in RGCs shown in (D). **p<0.01.

RGCs were analyzed from at least 7 non-adjacent retinal sections for each animal, from 3 mice per group.

(F) Representative images of flat-mounted retinas stained for RBPMS (red) and MARCKS (green). Note the markedly increased MARCKS staining in RGCs 2 weeks infected with AAV2-*Marcks* compared to that infected with AAV2-PLAP. Scale bar: 50 μm.

(G) Quantification of MARCKS fluorescence intensity in RGCs shown in (F). ***p<0.001.

RGCs were analyzed from at least 12 fields for each retina, from 3 mice per group.

**Figure S3. *Marcks* overexpression does not affect RGC survival.**

1. Timeline of the experimental procedures.
2. Representative images of flat-mounted retinas stained for RBPMS (white) to label

RGCs in the uninjured and injured for each condition 2 weeks after optic nerve crush. Scale bar: 50 μm.

1. Quantification of RGC survival in (B) showing that *Marcks* overexpression did not

affect RGC survival. ns=no significance. RGCs were analyzed from at least 7 fields for each retina, from 4 mice per group.

**Figure S4. *Marcks* overexpression does not change MARCKS phosphorylation levels in RGCs.**

1. Representative images of retinal sections showing that *Marcks* overexpression did

not change MARCKS phosphorylation levels in RGCs 3 days after optic nerve crush. Retinal sections were stained with RBPMS (red), and p-MARCKS (green). Scale bar: 20 μm.

1. Quantification of fluorescence intensity of p-MARCKS in RGCs shown in (A).

ns=no significance. RGCs were analyzed from at least 9 non-adjacent retinal sections for each animal, from 3 mice per group.

**Figure S5. *Marcks* overexpression in crushed RGCs does not significantly affect known regeneration-associated genes.**

1. Volcano plot of log_2_ (Fold-Change) versus –log_10_ (P.adjust) showing differences in

gene expression between *Marcks* overexpression and control conditions.

1. GSEA-GO analysis of RNA-seq data showing that *Marcks* overexpression did not

affect axon regeneration related gene transcription in RGCs.

1. Expression heatmap of selected genes known to promote (C) or restrict (D) axon

regeneration**.** Results showing *Marcks* overexpression did not significantly affect the transcription levels of these genes in RGCs.

**Figure S6. *Marcks* overexpression in naïve RGCs does not significantly affect General Gene Transcription in RGC.**

1. Principal component analysis of samples showing the high degree of similarity

in gene transcription between *Marcks* overexpression and control groups in naïve RGC.

1. Hierarchical clustering of samples showing the similarity in transcriptome between

*Marcks* overexpression and control groups in naïve RGC. The value in each grid represents the Euclidean distance between two samples.

1. Pairwise correlations of samples showing that *Marcks* overexpression had little

impact on gene transcription of RGCs. The upper right showed Pearson correlation coefficient between pairwise samples. The lower left gave their scatter plots of normalized counts.

1. GO analysis of differentially expressed genes between *Marcks* overexpression

and control groups showing that *Marcks* overexpression did not affect axon regeneration related gene transcription in uninjured RGCs.

**Figure S7. *Marcks* without ED domain does not promote axon regeneration.**

1. Top: experimental timeline. Bottom: representative image of optic nerve showing

that overexpression of *Marcks* without ED does not promote axon regeneration 2 weeks after optic nerve crush. The right columns showed enlarged images of nerves at 500μm distal to the crush sites marked by white boxes on the left. Scale bar: 200 μm. *: crush sites.

1. Quantification of axon regeneration in (A). ns=no significance. n=6 mice in each

group.

**Figure S1**


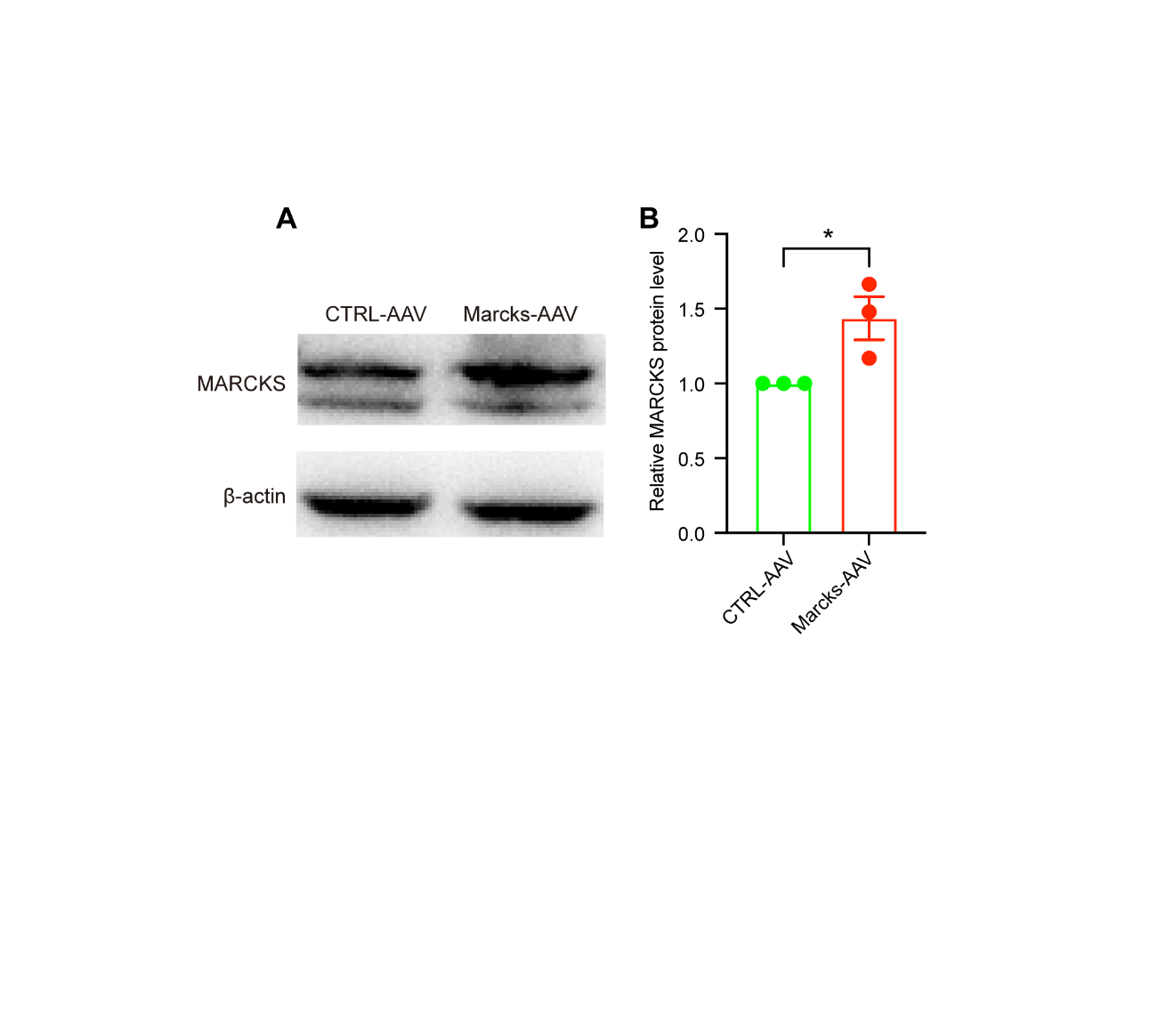


**Figure S2**


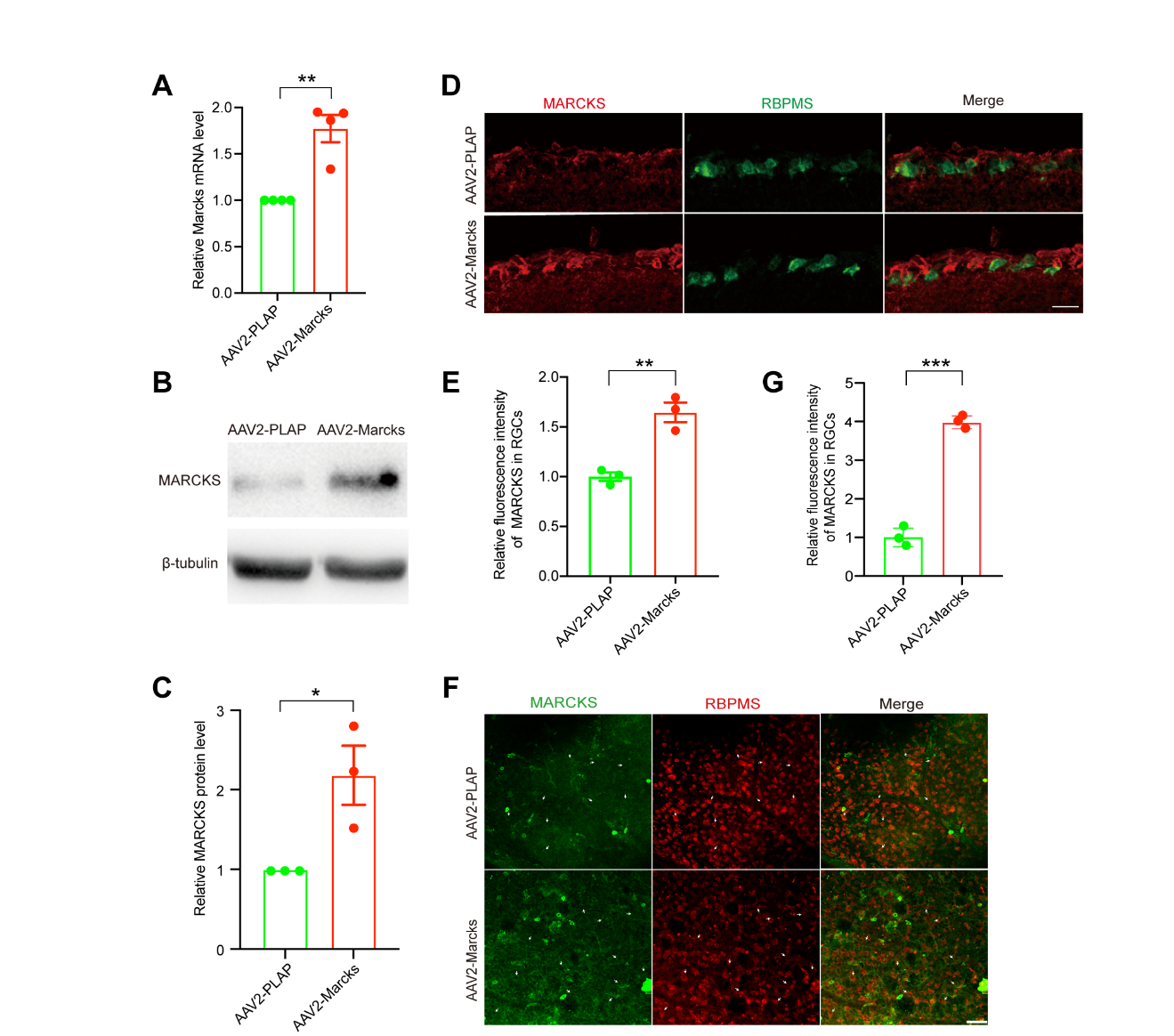


**Figure S3**


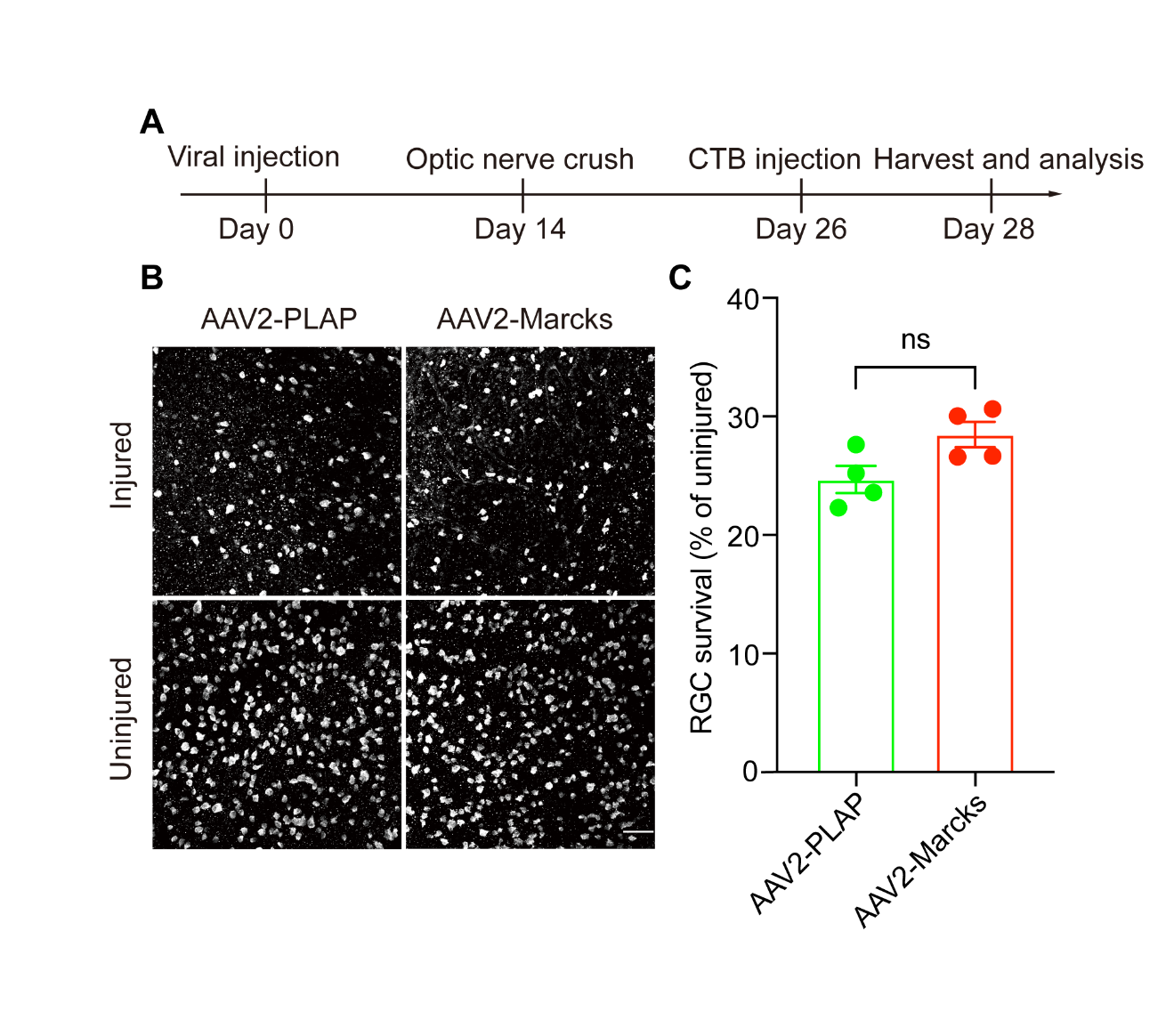


**Figure S4**


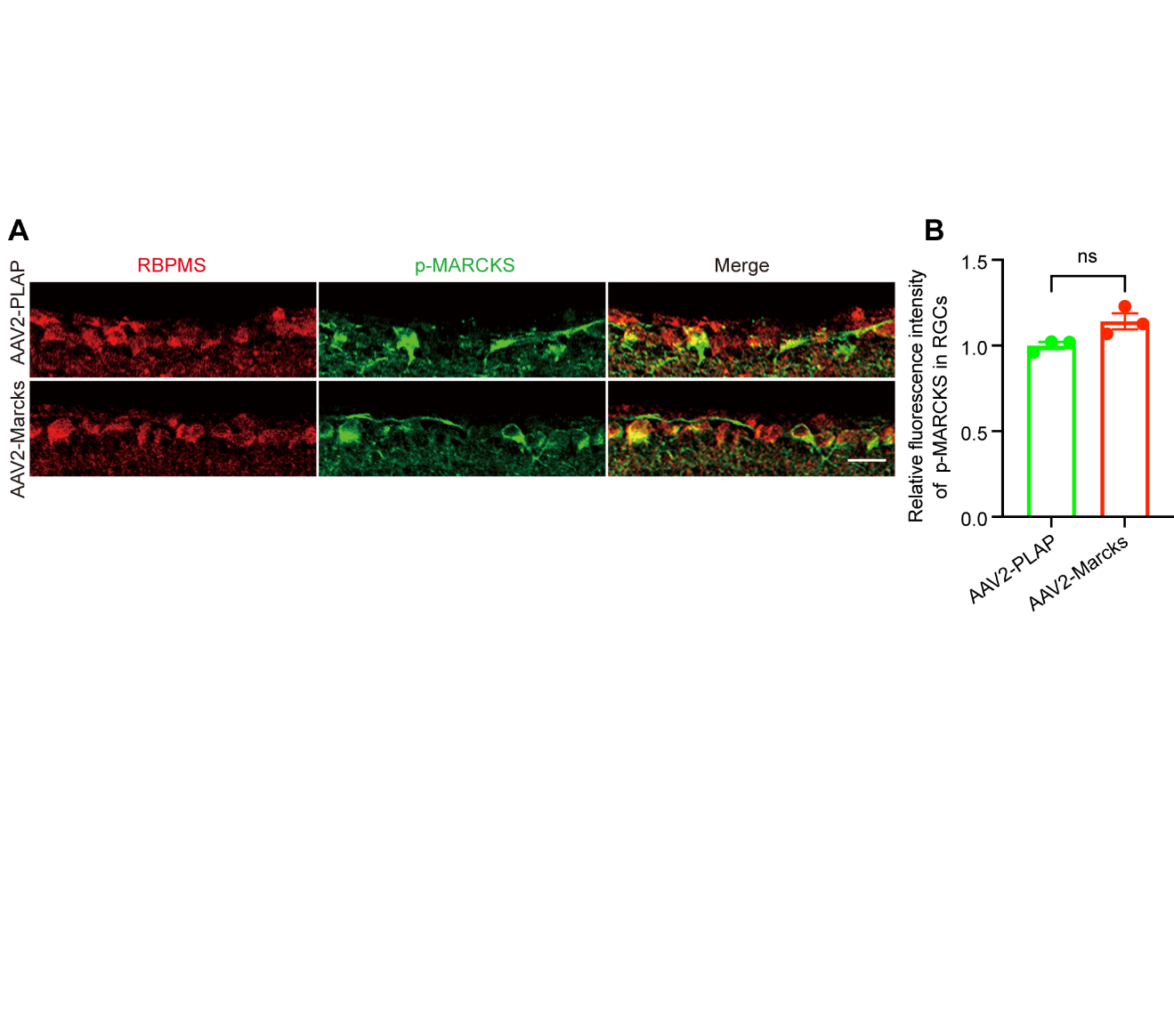


**Figure S5**


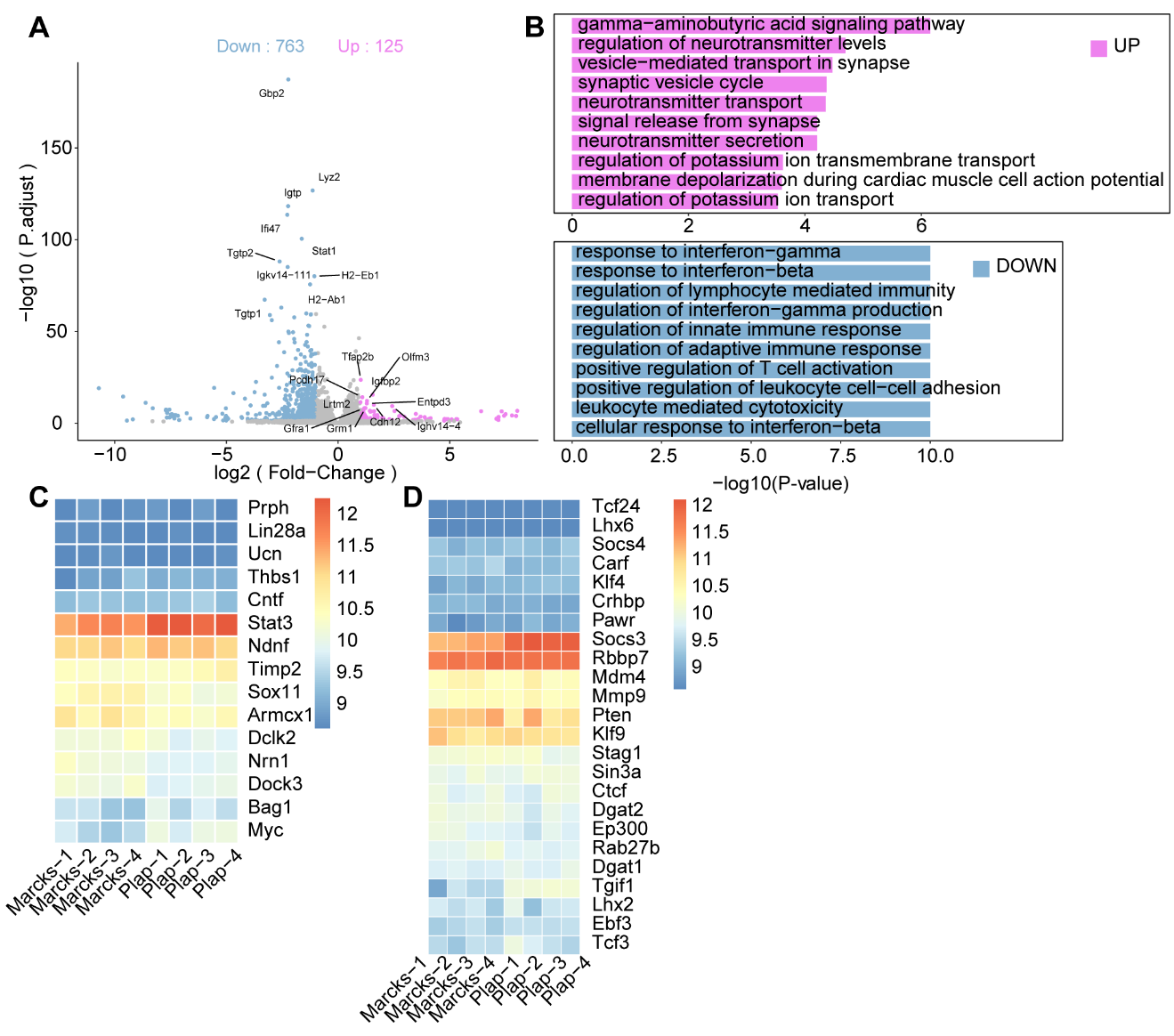


**Figure S6**


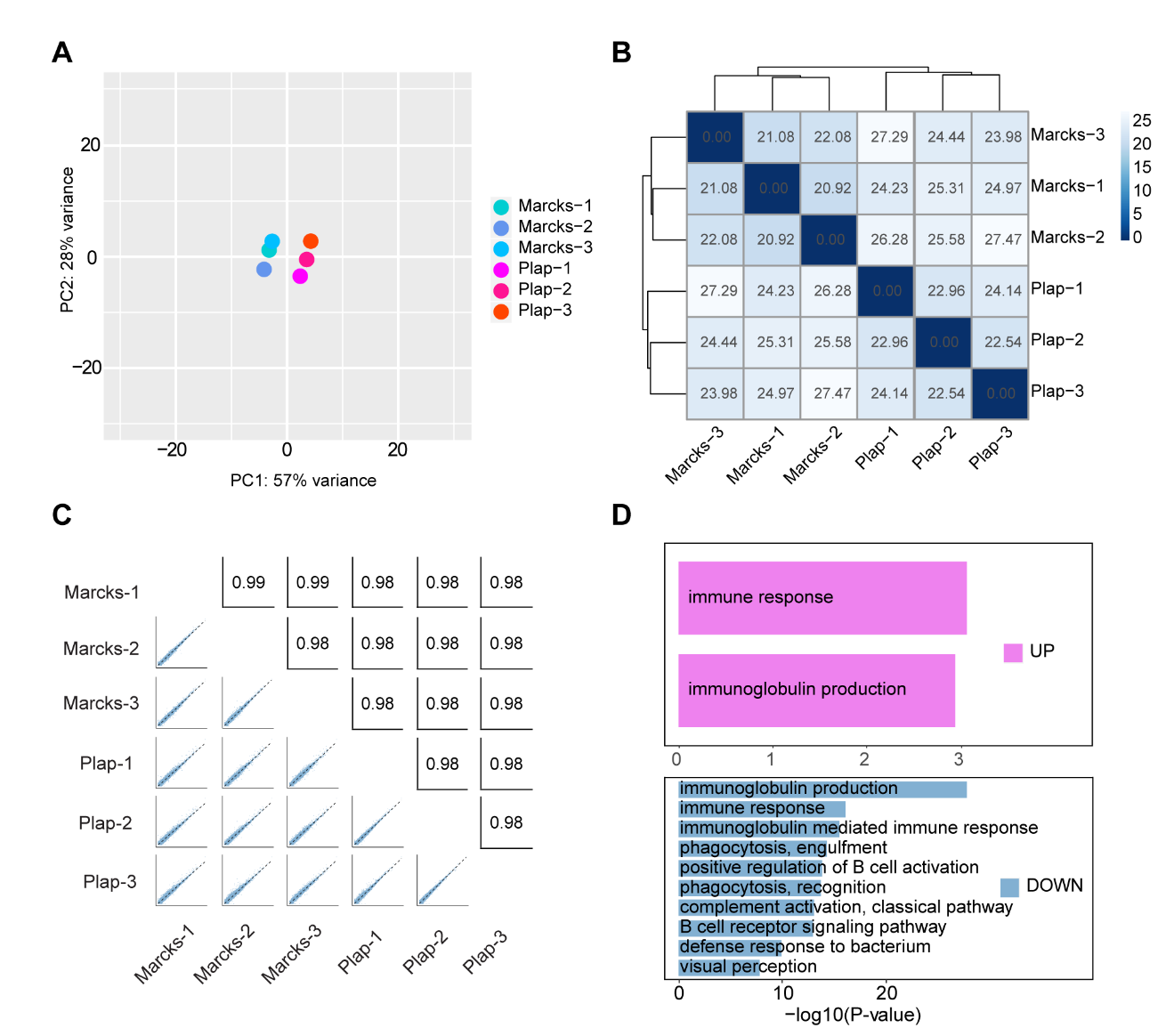


**Figure S7**


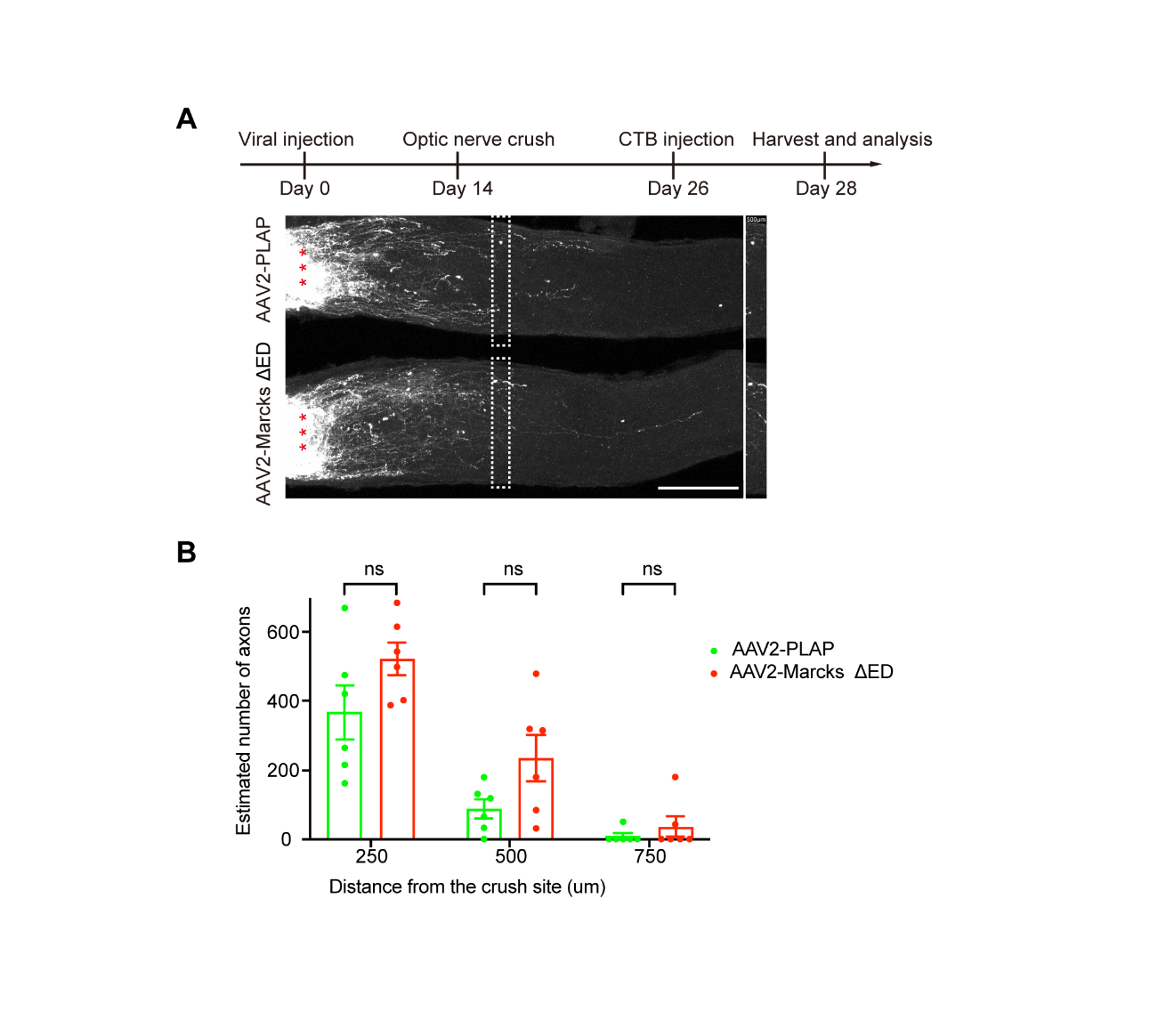

Supplement: Supplementary file 1 — Supplemental data [file 41419_2024_7281_MOESM1_ESM.docx]
