# Supplementary figures and images for: Marcks overexpression in retinal ganglion cells promotes optic nerve regeneration
Source: Cell Death Dis. 2024 Dec 18;15(12):906. doi: 10.1038/s41419-024-07281-6 (PMC11655864; doi:10.1038/s41419-024-07281-6)

## Slide 1
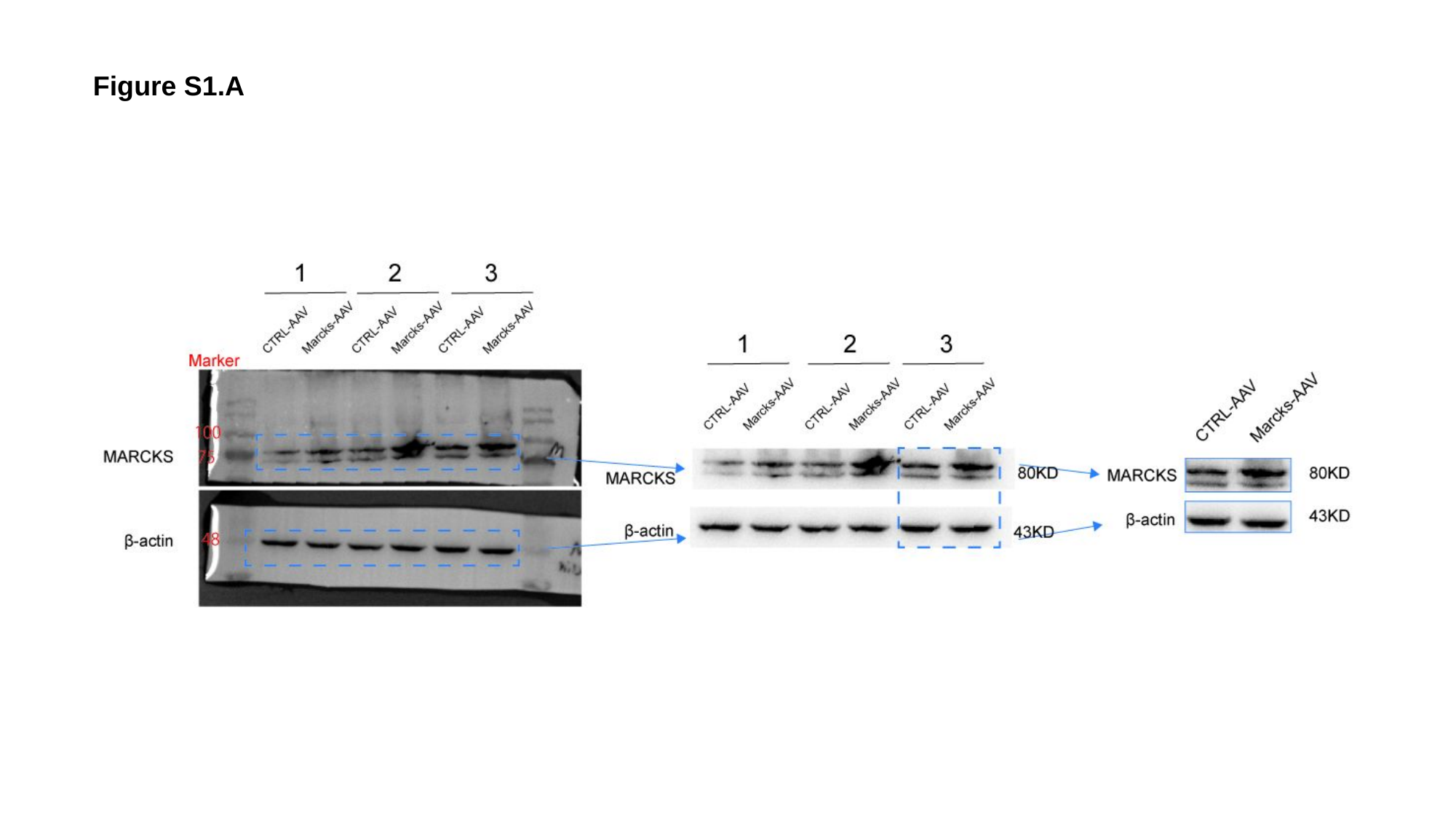

Figure S1.A

## Slide 2
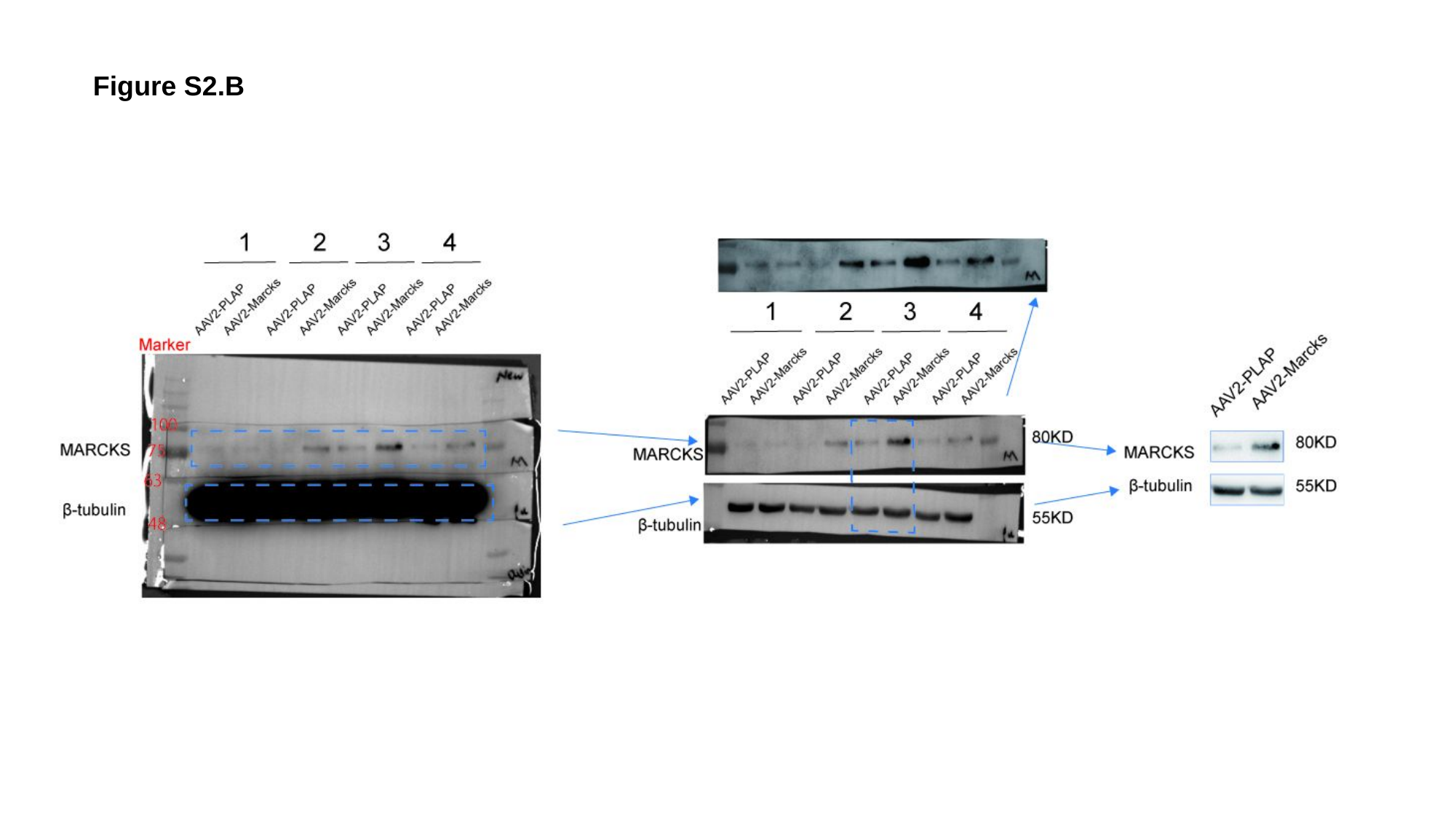

Figure S2.B

Supplement: Supplementary file 4 — Original Western blotting [file 41419_2024_7281_MOESM4_ESM.pptx]
